# Carry‐over effects of weather and decision‐making on nest success of a migratory shorebird

**DOI:** 10.1002/ece3.9581

**Published:** 2022-12-12

**Authors:** Sarah J. Clements, Jason P. Loghry, Bart M. Ballard, Mitch D. Weegman

**Affiliations:** ^1^ School of Natural Resources University of Missouri Columbia Missouri USA; ^2^ Caesar Kleberg Wildlife Research Institute Texas A&M University Kingsville Texas USA; ^3^ Department of Biology University of Saskatchewan Saskatoon Saskatchewan Canada

**Keywords:** climate, movement, reproductive success, shorebird, stopover, wader, weather conditions

## Abstract

Weather conditions experienced by birds can influence their migration decision‐making and strategy both within and across seasons. Additionally, decision‐making during migration may influence subsequent fitness (reproductive success and/or survival). Examining the effects of fine‐scale weather variables on individuals throughout the year could help identify stages of the annual cycle when species may be most affected by weather. In this study, we captured 24 black‐bellied plovers (gray plovers; *Pluvialis squatarola*) on nonbreeding areas along the western Gulf of Mexico coast and tracked their locations once every 2 h through their breeding season in the Alaskan and Canadian Arctic. We quantified migration strategies and weather conditions experienced by each individual throughout the nonbreeding, northward migration, and breeding seasons. We used a Bayesian hierarchical model which connected regressions linking weather with migration metrics, and migration metrics and breeding season weather with reproductive success. We found strong negative relationships between two migration metrics (migration duration and number of stopovers) and reproductive success, but no substantial relationships between breeding season weather variables and reproductive success. We found negative relationships between nonbreeding season temperature, migration temperature, and migration NDVI and both migration duration and number of stopovers, in addition to positive relationships between the number of stopovers and storms during migration, migration duration, and nonbreeding season precipitation. These results suggest that reproductive success is influenced by weather throughout the annual cycle and migration strategy is a key mechanism through which these effects operate. Our findings suggest that environmental factors throughout the year influence shorebird fitness, and, because black‐bellied plovers are often associated with mixed‐species flocks, many species likely experience similar constraints.

## Graphical abstract

Weather conditions experienced by birds can influence their migration decision‐making and strategy, which may affect subsequent fitness. We tracked black‐bellied plovers across three seasons and found relationships between weather and migration strategy, and between migration strategy and reproductive success. Our results suggest that reproductive success is influenced by weather throughout the annual cycle and migration strategy is a key mechanism through which these effects operate.

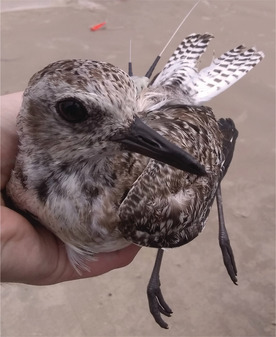

## 1 INTRODUCTION

Migratory shorebirds are threatened globally (Wauchope et al., 2017). In North America, shorebirds that migrate through the midcontinent may be at greater risk of decline than coastal populations (Thomas et al., 2006) due to the ephemeral nature of many midcontinent stopover sites and unpredictable resource availability (Skagen & Knopf, 1994). Shorebird decline has been linked to climate change (Galbraith et al., 2014; Piersma & Lindström, 2004). Research focused on phenological mismatch between shorebird migration or pulses of food and optimal timing of nesting has revealed variable patterns. For example, in a 7‐year study of seven species of Arctic‐nesting shorebirds, Weiser et al. (2018) found no evidence of effects of environmental variables or predator abundance at nest sites on reproductive traits. Smith et al. (2010) found that snowmelt and predators affected breeding chronology, while weather variables did not, and Saalfeld et al. (2019) found a positive relationship between shorebird chick growth and early arrival date, but no substantial relationship between chick growth and phenological mismatch due to interannual variability in weather. In other cases, conditions brought about by climate change can result in improved conditions for shorebirds in the short term, for example, due to decreased energy expenditure and increased food availability with warmer temperatures (Piersma & Lindström, 2004; van de Pol et al., 2010). However, over time, climate change is expected to negatively affect shorebirds and their habitats in all stages of the annual cycle (Galbraith et al., 2014; Piersma & Lindström, 2004), so environmental pressures from all seasons could be influencing shorebirds and should be studied.

Although it comprises a small part of the year, migration is an energetically costly and risky stage of the annual cycle (Piersma et al., 2016; Wilcove & Wikelski, 2008). Many studies on the effects of climate on shorebird reproductive success have focused on interannual variation at the population level and during the breeding season, but there may be carry‐over effects accumulating throughout the year which can influence fitness (i.e., survival and reproductive success) of individuals, and, therefore, the population. Carry‐over effects are events occurring in one season that bring about effects on individual fitness in a subsequent season (Harrison et al., 2011). Theoretical models and empirical studies have demonstrated that carry‐over effects can cascade to influence population dynamics (Inger et al., 2010; Norris, 2005). Examining the effects of fine‐scale variation in weather variables on individuals throughout the year provides a different perspective than an assessment of weather on demography. Unique yet complementary information can be provided by individual‐ and population‐ level studies. While population‐level studies often involve long‐term data sets with which we can attribute variation in demographic rates among years to environmental factors (e.g., Cunningham et al., 2021), individual‐level studies can result in detailed information about movement, behavior, and decision‐making, often over a relatively short period of time. Focusing at the individual level, we can identify drivers of animal decisions related to habitat use and migration strategy and relate these decisions to weather and landscape variables (e.g., Efrat et al., 2019; Senner et al., 2018). Individual tracking data can provide information about consequences of environmental characteristics on fitness through behavior and movement.

Weather conditions can influence many components of migration strategy, migration duration, route, and stopover decisions (Shamoun‐Baranes et al., 2010). European robins (*Erithacus rubecula*) were more likely to depart stopover sites when wind speed and precipitation were both low (Schaub et al., 2004). Season‐specific influences of wind and barometric pressure have been linked with golden eagles (*Aquila chrysaetos*) in Alaska taking a northern or southern route across mountains (Eisaguirre et al., 2018). However, for some species with long and risky migrations, characteristics related to migration phenology or strategy may not strongly influence fitness. Senner et al. (2014) suggested that the lack of carry‐over effects on timing of migration and reproductive success observed in Hudsonian godwits (*Limosa haemastica*) may be due to their extreme migration distance and transoceanic route causing mortality before carry‐over effects can take effect. Studying carry‐over effects of weather on migration strategy and fitness could help to identify stages of the annual cycle at which a changing climate is most likely to influence shorebird fitness.

**FIGURE 1:**
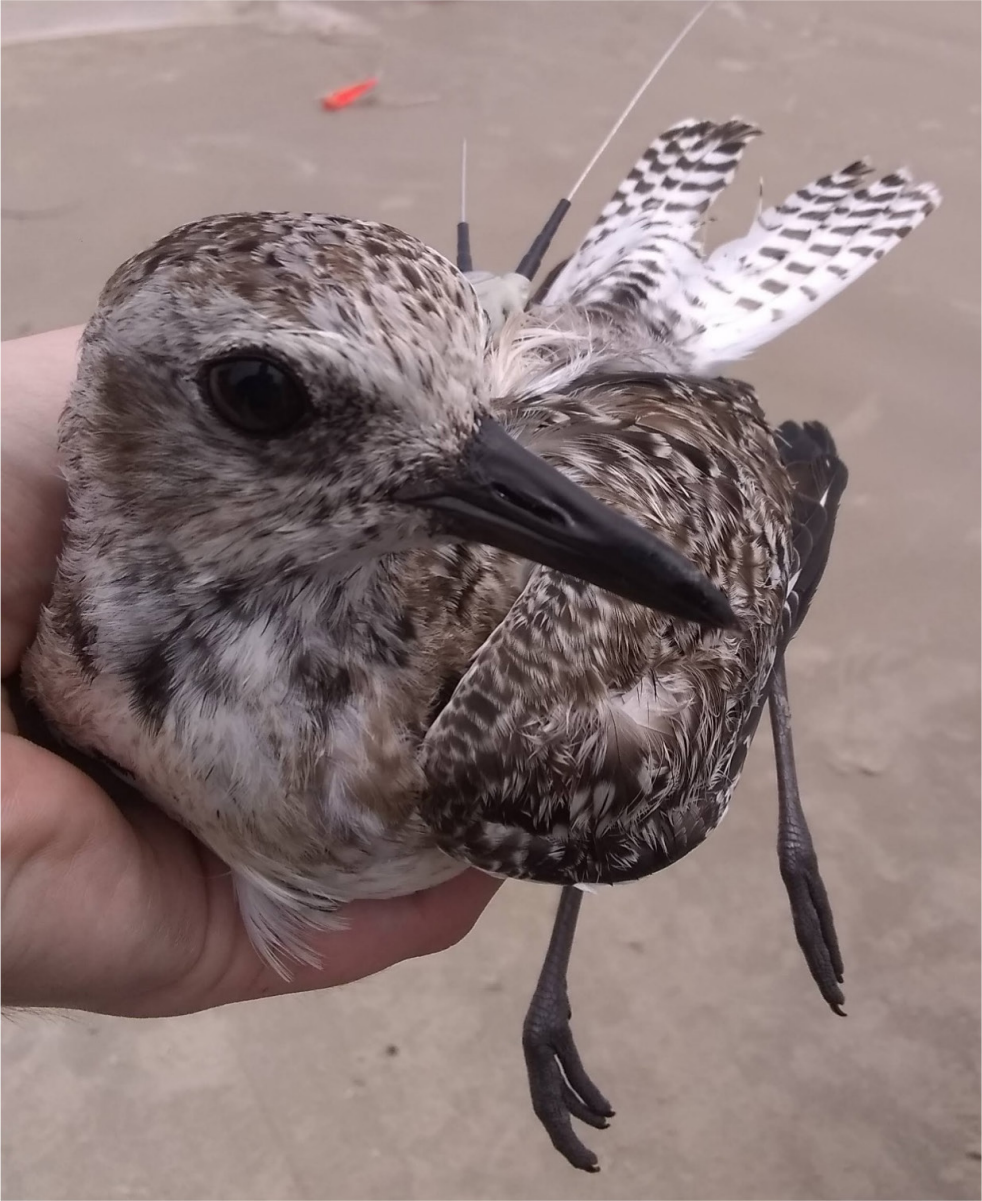
A black‐bellied plover captured in Texas in March 2021.

The black‐bellied plover (gray plover; *Pluvialis squatarola*, Figure 1) is a relatively large, Arctic‐nesting migratory shorebird that is distributed worldwide, and its association with mixed assemblages of shorebird species makes it a good model species for studies of shorebird ecology (Poole et al., 2020). After rapid decline between the 1970s and 1990s, populations in the western hemisphere appear to be more stable (Andres et al., 2012), but populations are still suspected to be in moderate decline in North America (Hope et al., 2019). In North America, most research on the species has focused on breeding ecology (Poole et al., 2020), so studying the nonbreeding season (winter in our study area) and spring migration adds to our understanding of black‐bellied plover ecology and more broadly, carry‐over effects on migratory shorebirds. The species is also widespread (Poole et al., 2020) so individuals have the potential to take different migration routes and choose different stopover and breeding locations. This variation, along with their associations with other species, makes them an appropriate species to study migration traits because findings can also be considered for other shorebird species that are too small to carry tracking devices that collect detailed GPS data.

Our objectives were to test for effects of nonbreeding (i.e., winter) and spring migration weather conditions on reproductive success through migration decision‐making in black‐bellied plovers, and examine effects of weather conditions during nonbreeding, migration, and breeding seasons on reproductive success. To meet these objectives, we quantified the effects of weather during the nonbreeding and breeding seasons on the number of stopovers and migration duration, and of number of stopovers, migration duration, and breeding season weather on reproductive success. We collated several variables representing weather conditions during nonbreeding and breeding seasons, including the Normalized Difference Vegetation Index (NDVI) as a proxy for food availability, temperature, extreme weather events, tailwinds, cumulative precipitation, and precipitation rate. We anticipated that higher NDVI at stopover sites around the time a bird was present would be associated with fewer or shorter stopovers because better conditions for invertebrates may increase foraging efficiency, as well as a shorter migration duration because if less energy is expended foraging during stopovers, birds may have more energy for migration. We also expected higher nonbreeding season and migration temperatures would be associated with shorter migrations and fewer stopovers because, similarly to NDVI, warmer temperatures could create favorable conditions for food availability. We expected that more extreme weather events during the breeding season would reduce the probability of reproductive success, and that extreme weather during the migration season would be associated with more stopovers and a longer migration duration, and that higher tailwind would be associated with shorter migration duration and fewer stopovers. We anticipated that cumulative precipitation and precipitation rate would decrease the number of stopovers and migration duration by causing more habitat to be available and allowing birds to efficiently locate and acquire nutrients, and that reproductive success would increase with breeding season precipitation. Finally, we expected that more efficient migration, with either fewer stopovers or shorter stopover durations, would be associated with increased probability of reproductive success.

## 2 METHODS

### 2.1 Study species

The black‐bellied plover is one of the most wide‐ranging shorebird species. In the western hemisphere, black‐bellied plovers spend the nonbreeding season in temperate and tropical areas of the Atlantic and Pacific coasts (Poole et al., 2020). Many individuals migrate through midcontinent North America. In coastal areas, their diet typically consists of invertebrates such as bivalves and polycheates (Poole et al., 2020). They migrate with relatively long steps between stopovers, and among our birds, time spent at individual stopover sites ranges from a few hours to over 9 days (Clements, 2022). They use freshwater habitat such as temporary wetlands, flooded fields, and the edges of lakes and reservoirs, in addition to occasional upland habitat, where they feed on primarily on invertebrates (e.g., insects, earthworms; Poole et al., 2020). Because habitat availability and distribution for black‐bellied plovers varies with environmental conditions (Skagen & Knopf, 1994; Steen et al., 2018) and phenology and abundance of invertebrates is linked to site productivity and conditions (Buchan et al., 2021; Pettorelli et al., 2005), we expected weather and environmental conditions to influence migration decision‐making and reproductive success.

### 2.2 Bird capture and deployment of tracking devices

**FIGURE 2:**
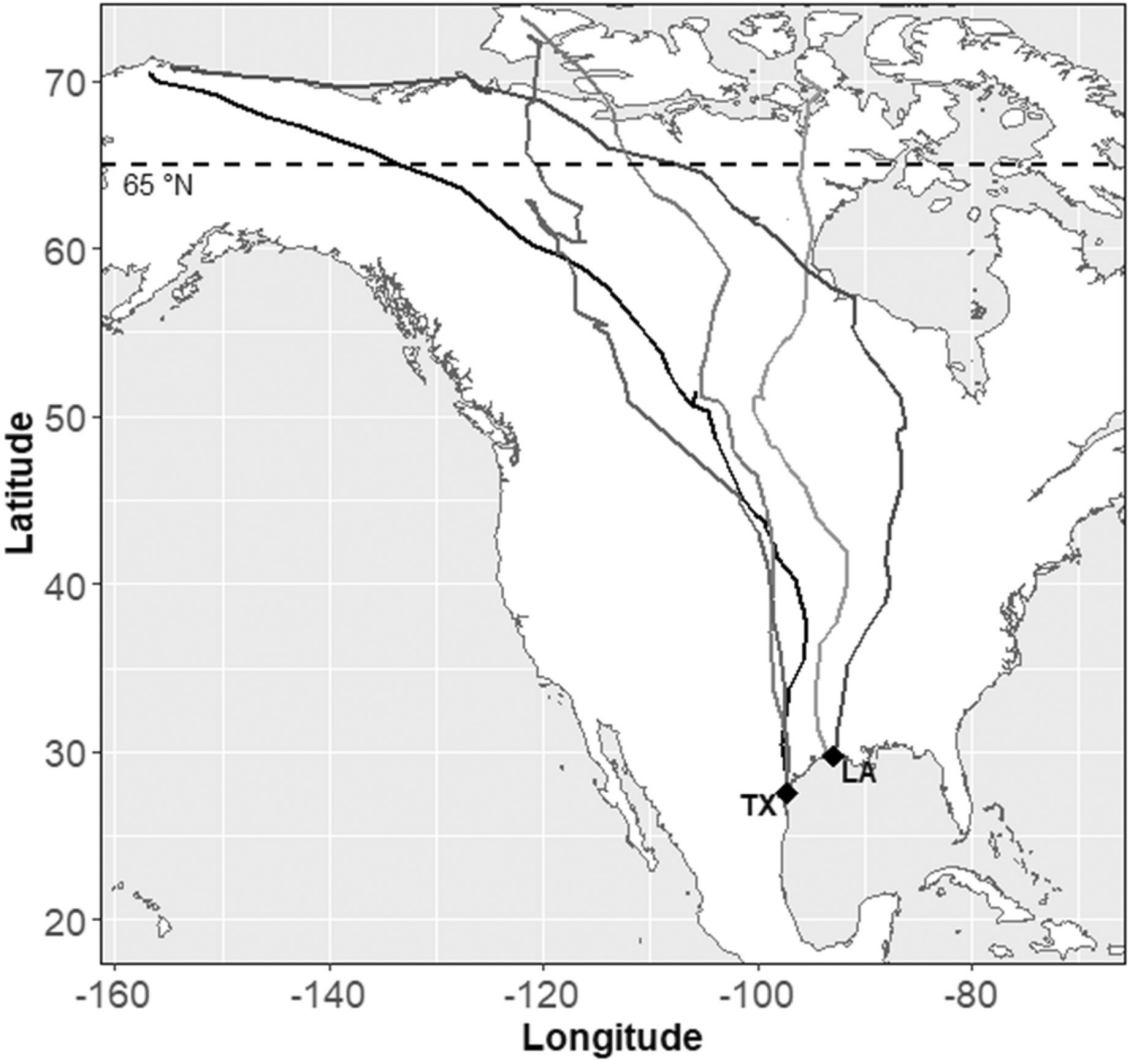
Five examples of black‐bellied plover spring northward migration tracks from our study; each track of an individual bird is represented in a shade of gray. Our capture sites in Texas (TX) and Louisiana (LA) are indicated by labeled points. Individuals migrated though the mid‐continent of North America to breeding areas often in the north slope of Alaska or Banks Island in the Canadian Arctic, but some breeding areas were farther east in Nunavut. The horizontal dotted line indicates 65°N; all breeding locations for birds in our study were above this latitude.

We captured black‐bellied plovers in coastal Texas and Louisiana, USA (Figure 2), using rocket nets and cannon nets. All birds captured were fitted with a USGS Bird Banding Laboratory metal band, under permit to B. Ballard (permit #21314). We aged birds according to molt and plumage characteristics (Meissner & Cofta, 2014). We collected morphometric data from each bird and a blood sample for molecular sexing (van der Velde et al., 2017). All captures took place during winter and spring (January–May) 2019–2021. Birds captured in Louisiana were individuals resident for the nonbreeding season and captured between early January and early March each year. Birds captured in Texas were captured in late March to early May and were presumed resident for the nonbreeding season, however, the few birds captured very late in the season could possibly have begun migration previously.

We deployed two models of tracking devices on black‐bellied plovers and ensured that the weight of the device was ~3% or less of bird body weight, but for this study we used only data from 6.6‐g Pinpoint Argos Solar S (*n* = 33) devices manufactured by Lotek (Newmarket, ON, Canada) due to their increased duty cycle capacity. We attached all devices using leg‐loop harnesses (Sanzenbacher et al., 2000) comprised of silicone (“Stretch Magic”) or elastic shock cord. The devices collected GPS data and uploaded it via the Argos satellite system (Woods Hole Group). Devices were programmed to collect a GPS fix every 2 h (12 locations per day) and the GPS data they collect is precise to ±10 m (Clements et al., 2022). Devices were expected to last from deployment through the breeding season, and only devices which collected data consistently through 15 July of the year they were deployed were included in analyses to ensure that data were collected through expected end of incubation in mid‐July. Twenty‐four of 33 devices collected data at least through 15 July and covered the entire migration period, and two of these appeared to have recorded a mortality event or a detachment of the device from the bird. The remaining nine devices which did not collect data through 15 July appeared to be abrupt failures, so we were not able to determine fate of birds.

### 2.3 Tracking data storage and processing

Data from devices were stored in Movebank (Kranstauber et al., 2011). We used Movebank to filter location quality based on Cyclic Redundancy Check status for Lotek tags. Then, we used the SDLFilter package (Shimada et al., 2012) in Program R to remove duplicate points and outliers based on a speed threshold of 150 km/h (Linscott et al., 2022; Shimada et al., 2012). The SDLFilter package calculates speed based on GPS locations and timestamps and can also detect duplicate points in space and time. Finally, we visually identified and removed several remaining outlier points, most of which showed erroneous future times or were located at 0° latitude and longitude.

### 2.4 Identifying reproductive success

We used full‐term incubation (24 days) as a proxy for reproductive success because it was most identifiable using GPS data. A full‐term incubation for black‐bellied plovers is 24–26 days and males and females share incubation duties, switching every few hours (Poole et al., 2020). Incubation time is expected to vary slightly by sex (male incubates more toward the beginning, female more toward the end; Poole et al., 2020). All black‐bellied plovers used in this analysis were identified as male via molecular sexing. Thus, we expected birds incubating a clutch to have successive points in a relatively small area for the full term of incubation. Our criteria for ascribing full‐term incubation were based on average daily displacement and patterns of return to a potential nest site. If a bird showed daily average displacement of <500 m for the duration of the incubation period (i.e., average displacement between points each day remained below 500 m), a stationary site was visually identifiable, and the individual consistently returned to the area within a 30‐m buffer around a potential nest site for 24 days, that bird was ascribed as having a successful nest that year.

Data post‐incubation period (i.e., from August) were also used for devices presumed dropped or on dead individuals (devices remaining stationary past the expected end of incubation) to ensure we would not incorrectly ascribe breeding status to these individuals. Two individuals were assigned NA (i.e., unknown) for breeding because they were stationary during and after the incubation period. This indicated a dropped device or mortality sometime after incubation had started, so we were not able to discern breeding status. We recaptured and resighted black‐bellied plovers with their leg bands (confirmed or presumed to have been banded as part of our study) during the subsequent fall and winter after spring tagging, but did not see devices on some individuals in both Texas and Louisiana (J. Loghry & S. Clements, personal observation). Thus, there is evidence that devices were dropped between deployment and the subsequent winter. Therefore, we chose to assign these two individuals as NA rather than unsuccessful nesters.

### 2.5 Identifying stopover sites

We first identified points within the breeding range using a 50% kernel density estimate on all points above 65° latitude. We chose this latitude because all migrations in our study led to breeding sites north of this latitude and it corresponds approximately with the transition to the tundra (Figure 2). We defined the last point of migration as the last point collected before the bird crossed into the breeding area. We then identified stopover sites as points < 30 km from a previous point. A threshold of 30 km was chosen based on the distribution of step lengths between points and visual inspection of point clusters. Exo et al. (2019) used a similar displacement‐based approach to identify black‐bellied plover stopover sites in the East Atlantic Flyway using a 20‐km distance threshold, however we chose 30 km because it resulted in fewer subdivisions of stopover sites by a single long movement. After identifying whether or not each point was less than 30 km in distance from the subsequent point, we used run length encoding to identify clusters. We applied the following criteria to identify stopover sites: (1) ≥3 consecutive points within 30 km of one another or at least 6 h within the area, and (2) not within wintering or breeding areas. We chose to classify both short and long stopping periods as stopovers because birds may stop over for a variety of purposes that may influence their decision‐making and fitness (Linscott & Senner, 2021), and our high data resolution allowed us to identify stopover events that would not be detectable with less data. We then visually identified stopover sites and in a few cases where a bird left a stopover site briefly (one point) and later returned we ascribed that area as one stopover site. Each stopover site was assigned a unique label. We ascribed the nonbreeding area as the first such cluster of points in the data set, such that that the first point of migration was the first movement over 30 km from the nonbreeding area that led to the eventual crossing into the breeding area (Soriano‐Redondo et al., 2020). We used the first cluster of points for nonbreeding areas because unlike breeding areas in which birds are moving and searching for foraging and nesting habitat, our black‐bellied plovers remained at the same site throughout the nonbreeding season (i.e., the nonbreeding area would be classified as a stopover site under our framework for stopover identification). A summary of definitions of stages of the annual cycle for this analysis is provided in Table 1.

**TABLE 1:** Description of terms used to characterize the annual cycle in the context of our data analysis.

Term	Definition
Breeding season	Locations collected north of 65°N between June 15th and July 15th
Non‐Breeding season	Locations collected between bird capture and the first movement of over 30 km between points
Migration	All points after the first movement of over 30 km that led to the eventual crossing of the breeding area and the point at which the breeding area is crossed (we focus on northward migration only)

We calculated two migration metrics to characterize individual decision‐making. First, we counted all unique stopover sites used by each bird (NSTOP) and then calculated migration duration as the amount of time between the first and last GPS locations classified as migration points (MGDR). Number of stopovers and duration of migration are expected to influence fitness because of the importance of balancing energy acquisition and expenditure during migration (e.g., Gómez et al., 2017; Prop et al., 2003). We chose these metrics because number of stopovers can be thought of as related to energy acquisition and stopover sites are important considerations for land management strategies, while migration duration may be more related to the balance between energy acquisition and expenditure and is driven by migration phenology, which is important to monitor as environmental cues may change in the future. These terms were not strongly correlated (*r* = 0.14). We did not expect them to be highly correlated in black‐bellied plovers due to the high variation in stopover duration and the long migratory steps the birds often take between stopover events (Clements, 2022).

### 2.6 Weather data

We acquired temperature and precipitation data for the nonbreeding, migration, and breeding seasons; barometric pressure for the migration and breeding seasons; and Normalized Difference Vegetation Index (NDVI; a proxy for food availability) for the migration season to test hypotheses about drivers of migration metrics. Using data from all seasons help to understand the relative effects of conditions throughout the winter when birds are preparing for migration and conditions during the migration period. We extracted weather separately for each season because weather metrics may influence birds differently in each season. Additionally, local weather conditions can be more important than broad‐scale climate patterns for migration decisions (Tøttrup et al., 2010), so we aimed to summarize each variable in ways that took advantage of the spatial variation we were able to capture with spatial data, while providing an index of overall conditions throughout each season.

#### 2.6.1 NDVI

NDVI indicates net primary productivity, which influences availability of insects (Buchan et al., 2021; Sanz et al., 2003). Black‐bellied plovers feed on invertebrates; primarily insects during breeding and migration periods when they are away from coastal habitats (Poole et al., 2020). We downloaded 16‐day, 1‐km resolution NDVI data (Didan, 2015) using the AppEEARS web application (appeears.earthdatacloud.nasa.gov) and used data collected in May and June of 2019–2021 (ordinal days 129, 145, 161, and 177). We extracted the average NDVI within a 30 km buffer around each point within a stopover for the point in time closest to the time at which the GPS location was collected. We then summarized NDVI for the entire migration by calculating the mean NDVI for all locations at times each bird was there.

#### 2.6.2 Temperature

We acquired surface temperature data from the National Centers for Environmental Prediction using the RNCEP package (Kemp, Shamoun‐Baranes, et al., 2012; Kemp, van Loon, et al., 2012). We downloaded temperature data from the NCEP Reanalysis 1 interpolated to each bird's set of locations in space and time using RNCEP. We then calculated mean temperature across all migration points and breeding points identified (see above). Because we had variable amounts of winter tracking data among birds and our captures were concentrated at two specific nonbreeding sites in Louisiana and Texas, we extracted nonbreeding temperature data from the months of January through April from the grid cells in which the sites were located for 2019–2021.

#### 2.6.3 Wind

Similarly, we downloaded wind data using RNCEP for all migration points that were categorized as flight (i.e., not a stopover, breeding, or nonbreeding point as described in “Identifying Stopover Sites” above). We used RNCEP to calculate flow assistance (i.e., tailwind; Kemp, van Loon, et al., 2012; Kemp, Shamoun‐Baranes, et al., 2012) and used the mean flow assistance at flight points to represent wind experienced by birds during migration.

#### 2.6.4 Severe weather

We also downloaded barometric pressure from RNCEP as an indicator of severe weather. We used rasters of barometric pressure data from January to July of 2019–2021 based on a polygon encompassing the entire range of the black‐bellied plovers in our study during this time period. We then summarized barometric pressure for each day in each location the bird was in during the migration and breeding seasons. We calculated daily barometric pressure and change in barometric pressure since the previous day. Then, we classified the lowest 10% of barometric pressure as low pressure and the highest 10% of change in barometric pressure as a large drop in pressure. If the day a bird was located at a point was classified as both low pressure and a large drop, we assumed the bird experienced severe weather. We counted the number of unique days a bird experienced severe weather during migration and breeding. Extreme weather events may decrease prey density (Corte et al., 2017) and during the migration season extreme weather is known to result in in‐flight mortality for birds (Newton, 2006), so it is reasonable to suspect that some effects such as forced stopovers or re‐routing of movements may occur. During the breeding season, extreme weather can also affect productivity in birds (Martin et al., 2017).

#### 2.6.5 Precipitation

We downloaded precipitation data from the NOAA Climate Prediction Center for the years of 2019–2021. For precipitation in breeding areas, we calculated mean daily precipitation across grid cells in which breeding season GPS locations for each bird were located between the first day of the year and the end of July during the year the bird nested. For nonbreeding precipitation, we calculated mean daily precipitation across grid cells in which the bird's wintering site was located from January through April of the year the bird was captured. For migration, we calculated cumulative mean daily precipitation from early winter until mid‐May across all stopover sites, as the effect of spring precipitation on the presence of shorebirds can be influenced by previous precipitation (J. Jorgensen, R. Penner & M. Hanan, personal observation). We also calculated the precipitation rate at each point collected for a bird in the 5 days leading up to the day it was at each location, because available water at stopover sites in the Great Plains can be dynamic and vary quickly in time and birds may opportunistically select stopover habitat.

### 2.7 Hierarchical model

We hierarchically modeled the effects of nonbreeding and migration weather on migration metrics, and the effects of migration metrics and breeding season weather on reproductive success (i.e., full‐term incubation). We calculated pairwise correlations between all weather covariates and did not include any two with *r* > 0.50 within the same level of the model (Table 2). The hierarchical model allowed for uncertainty to propagate though all levels of the model. We used a single model comprised of variables based on our ecological questions rather than a model selection approach because we wanted to compare the effects of individual variables to one another within the model rather than comparing the fit of competing combinations of variables. We ran the model in Jags (Plummer, 2003) through Program R version 4.1.2 (R Core Team, 2021) using the JagsUI package (Kellner, 2015). We determined convergence based on R‐hat <1.05 (Brooks & Gelman, 1998) and visual inspection of chains. We also included posterior predictive checks and evaluated posterior predictive p‐values using JagsUI (Kellner, 2015). The posterior predictive *p*‐value is based on the discrepancy between the predicted and real data; a value close to 0.5 indicates that the model and data are consistent with one another, while a value close to 0 or 1 indicates that the predicted and original data are inconsistent with one another (Gelman et al., 1996; Kacker et al., 2008). Model code is provided in the Appendix S1.

**TABLE 2:** Pairwise correlation coefficients (*r*) between weather covariates in the hierarchical model (in order: tailwind, NDVI, breeding storms, migration storms, migration temperature, breeding temperature, winter temperature, breeding cumulative precipitation, migration cumulative precipitation, winter cumulative precipitation, and spring precipitation rate).

	TWDm	NDVI	STb	STm	TPm	TPb	TPw	PRCb	PRCm	PRCw	PRRm
TWDm	1.0	0.19	−0.28	0.15	−0.02	0.00	0.00	0.07	0.13	−0.03	0.21
NDVI		1.00	0.08	−0.28	0.05	0.40	0.26	0.27	0.19	0.13	0.26
STb			1.00	−0.17	0.26	0.46	−0.18	0.20	0.35	0.28	0.00
STm				1.00	−0.41	−0.06	−0.43	−0.14	−0.35	−0.02	0.09
TPm					1.00	−0.08	0.24	0.23	0.27	−0.06	−0.10
TPb						1.00	−0.19	0.23	0.15	0.47	−0.03
TPw							1.00	0.39	0.45	0.13	−0.28
PRCb								1.00	0.53	0.28	−0.10
PRCm									1.00	0.47	0.04
PRCw										1.00	−0.05
PRRm											1.00

*Note*: Values over 0.40 are highlighted in gray.

Our hierarchical model was comprised of two linear regressions and one logistic regression,

$${\text{NSTOP}}_{i}=\beta_{0}^{\left[\text{NSTOP}\right]}+\beta_{1}{\mathrm{STm}}_{i}+\beta_{2}{\text{NDVIm}}_{i}+\beta_{3}{\mathrm{TPw}}_{i}+\beta_{4}{\mathrm{TPm}}_{i}+\beta_{5}{\text{PRCm}}_{i}+\beta_{6}{\text{PRRm}}_{i}$$

$${\text{MGDR}}_{i}=\beta_{0}^{\left[\text{MGDR}\right]}+\beta_{7}{\text{TWDm}}_{i}+\beta_{8}{\text{NDVIm}}_{i}+\beta_{9}{\mathrm{TPw}}_{i}+\beta_{10}{\mathrm{TPm}}_{i}+\beta_{11}{\text{PRCw}}_{i}$$

$$\text{logit}\left({\text{BREED}}_{i}\right)=\beta_{0}^{\left[\text{BREED}\right]}+\beta_{12}{\text{NSTOP}}_{i}+\beta_{13}{\text{MGDR}}_{i}+\beta_{14}{\mathrm{TPb}}_{i}+\beta_{15}{\mathrm{STb}}_{i}+\beta_{16}{\text{PRCb}}_{i}$$

where $\beta_{0}$ was the intercept in each regression, and for each individual bird *i*, *NSTOP* was the number of stopover sites, *MGDR* was the migration duration in days, *BREED* was a binary variable with 1 for successful full‐term incubation, 0 for failed incubation or deferral of reproductive attempt, and NA if breeding status was not able to be determined. *STm* and *STb* were the number of days during migration and breeding, respectively, that the bird experienced extreme weather (i.e., strong storms). *NDVIm* was the mean NDVI across all migration points. *TPw*, *TPm*, and *TPb* were the mean temperatures across the nonbreeding, migration, and breeding seasons, respectively. *PRRm* was the mean precipitation rate within the 5 days leading up to each migration point. *PRCw* was the cumulative precipitation for each year in nonbreeding locations, and *PRCm* and *PRCb* were cumulative precipitation for each year in migration and breeding locations, respectively. *TWDm* was the mean flow assistance (tailwind) for flight points during migration. We report the posterior mean for each β and 95% credible interval, as well as the proportion of posterior samples that were >0 or <0 on the same side as the mean (*P*) as evidence that the β was positive or negative, for each regression. We plotted predicted values of the relationship between a response and an effect when the proportion of posterior samples above or below 0 (*P*) was >0.80.

## 3 RESULTS

The model was run using information for 24 birds. The number of stopovers for an individual ranged from 2 to 10 (mean = 6.17, standard deviation [SD] = 1.73). Migration duration ranged from 17.08 to 32.91 days (mean = 23.39, SD = 4.46). Eleven black‐bellied plovers were classified as successful in full‐term incubation, 11 were unsuccessful, and two were unknown. Summaries of each weather variable are presented in Table 3. Posterior predictive *p*‐values for each level of the hierarchical model were 0.50 for the number of stopovers and migration duration regressions, and 0.48 for the breeding regression, indicating that the model predicted consistently with the real data.

**TABLE 3:** Summary of weather covariate data calculated across each season of interest, migration tailwind (TWDm), migration NDVI (NDVI), migration storms (STm), breeding storms (STb), migration temperature (TPm), breeding temperature (TPb), winter temperature (TPw), winter cumulative precipitation (PRCw), migration cumulative precipitation (PRCm), and migration precipitation rate (PRRm).

Covariate	Mean	Min	Max	SD
TWDm (m/s)	1.95	0.21	3.58	0.87
NDVI	0.36	0.27	0.48	0.06
STm	1.75	0.00	4.00	1.11
STb	0.83	0.00	4.00	0.92
TPm (K)	289.83	286.22	293.18	1.98
TPb (K)	277.92	275.00	284.42	2.19
TPw (K)	287.29	286.00	290.43	1.62
PRCw (cm)	415.13	184.17	655.00	140.97
PRCm (cm)	84.48	36.14	216.54	44.07
PRRm (g/m^2^/s)	0.003	0.001	0.007	0.002

**FIGURE 3:**
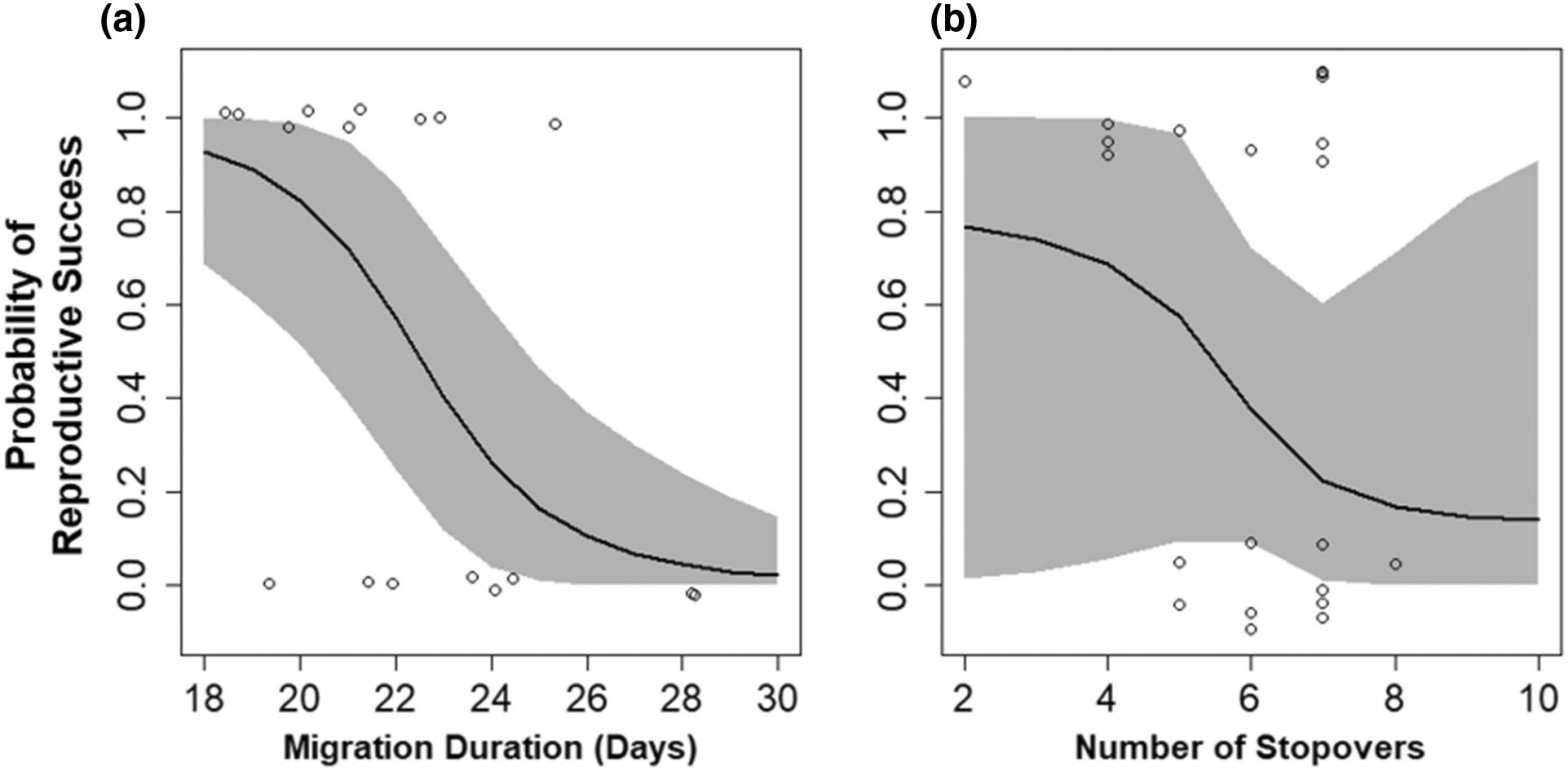
Predicted effect of (a) migration duration and (b) number of stopovers on black‐bellied plover breeding success. The line represents the mean, the gray ribbon represents the 95% credible interval, and the points represent raw data.

Migration duration was a stronger predictor of reproductive success (*β* = −0.77, 95% CRI −1.56, −0.25; *P* = 1.00) than the number of stopovers (*β* = −0.85, 95% CRI −3.31, 0.83; *p* = .85), but both effects were negative and explained substantial variation in reproductive success (Figure 3). Of the breeding season weather metrics we tested, temperature (*β* = 0.02, 95% CRI −0.19, 0.26; *P* = .60), cumulative precipitation (*β* = 0.00, 95% CRI −0.06, 0.06; *P* = .51), and number of storm days (*β* = −0.04, 95% CRI −2.09, 2.13; *P* = .53) did not explain substantial variation in breeding success.

**FIGURE 4:**
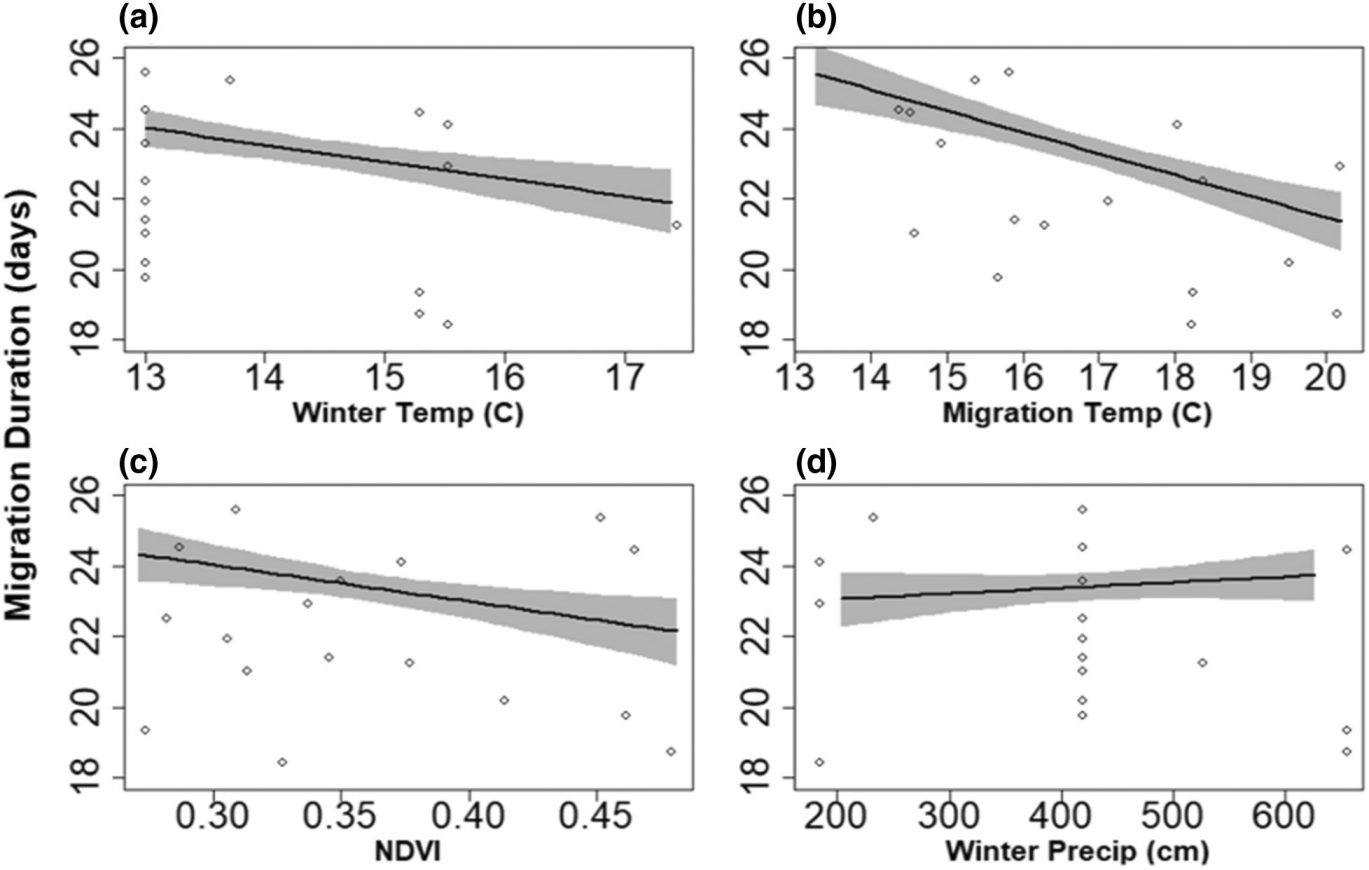
Predicted effect of (a) winter (nonbreeding) temperature, (b) migration temperature, (c) NDVI, and (d) winter cumulative precipitation on migration duration (days) in black‐bellied plovers. The lines represent the mean, the gray ribbons represent the 95% credible interval, and the points represent raw data.

Most weather metrics we tested explained substantial variation in migration duration except tailwind (*β* = 0.06, 95% CRI −0.34, 0.48; *P* = .60). Strong relationships between migration environmental variables and migration duration included NDVI (*β* = −0.63, 95% CRI −1.04, −0.20; *P* = 1.00), and migration temperature (*β* = −1.19, 95% CRI −1.62, −0.76; *P* = 1.00). NDVI and migration temperature had negative relationships with migration duration (Figure 4). Strong winter weather effects on migration duration included temperature (*β* = −0.78, 95% CRI −1.22, −0.34; *P* = 1.00) and cumulative precipitation (*β* = 0.22, 95% CRI −0.18, 0.67; *P* = .85). Nonbreeding temperature had a negative relationship with migration duration while nonbreeding precipitation had a positive relationship with migration duration (Figure 4).

**FIGURE 5:**
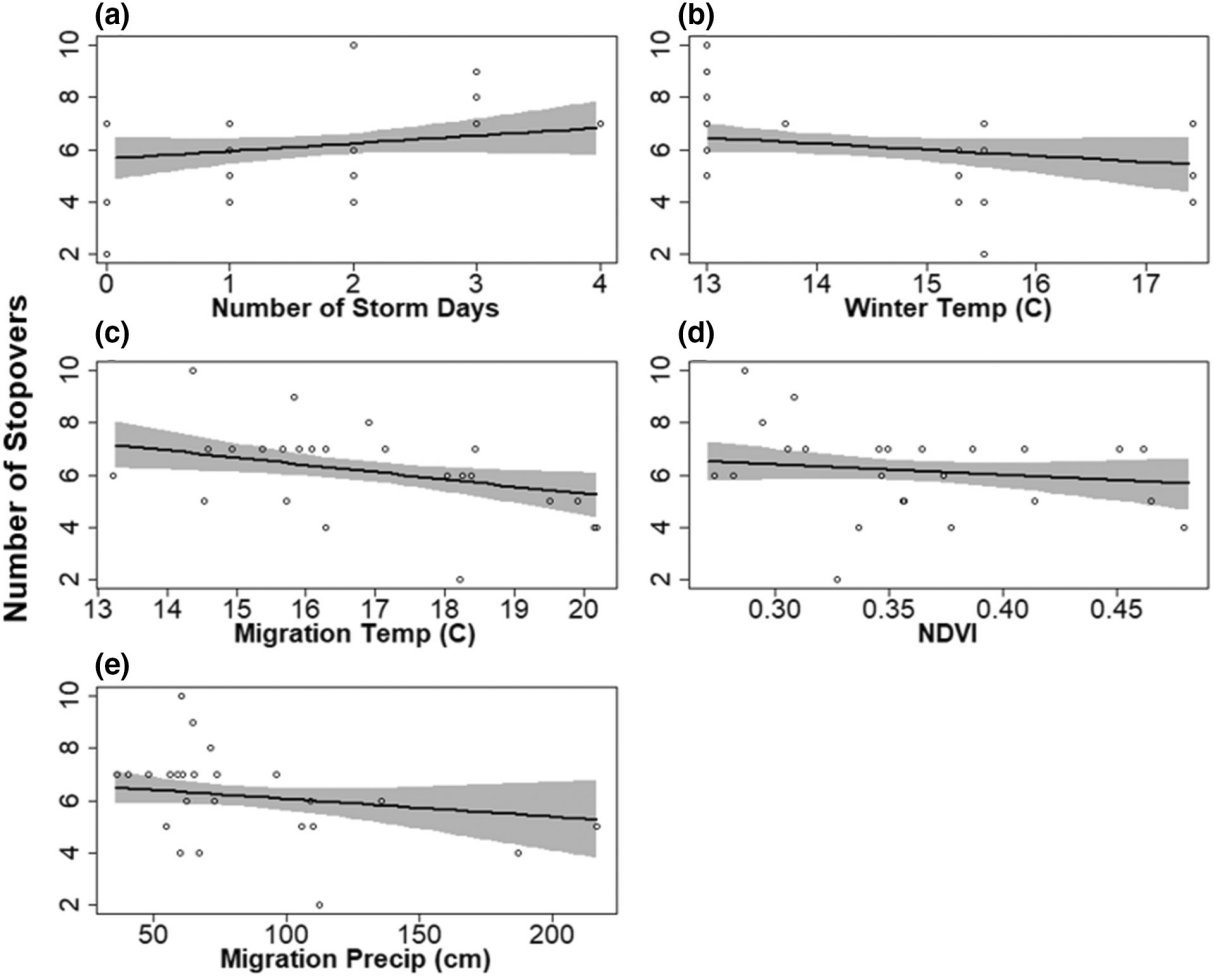
Predicted effect of (a) number of days with a storm, (b) winter temperature, (c) migration temperature, (d) NDVI, and (e) migration cumulative precipitation on number of stopovers during spring migration in black‐bellied plovers. The lines represent the mean, the gray ribbons represent the 95% credible intervals, and the points represent raw data.

Most weather metrics we tested also explained substantial variation in the number of stopovers. Migration weather variables for which *P* > 0.8 included number of storm days (*β* = 0.33, 95% CRI −0.14, .81; *P* = .91), NDVI (*β* = −0.25, 95% CRI −0.70, 0.20; *P* = .87), temperature (*β* = −0.55, 95% CRI −0.10, −0.09; *P* = .99), and cumulative precipitation (*β* = −0.30, 95% CRI −0.77, 0.16; *P* = .91). Nonbreeding temperature (*β* = −0.38, 95% CRI −0.89, 0.14; *P* = .93) also explained variation in the number of stopovers. The relationship between number of stopovers and migration precipitation rate was weak (*β* = 0.12, 95% CRI −0.32, 0.57; *P* = .81). The number of storms had a positive relationship with the number of stopovers, while NDVI, migration temperature, migration cumulative precipitation, and nonbreeding temperature had negative relationships with the number of stopovers (Figure 5).

## 4 DISCUSSION

We found that migration duration and number of stopovers were negatively related to reproductive success, and that weather variables during both migration and nonbreeding influenced these two migration metrics. These results suggest that decision‐making during migration is important for reproductive success and is also a mechanism through which environmental conditions throughout winter and spring can influence breeding season performance. With the onset of rapid climate change, weather conditions will likely become influential in the future and these results add to the current understanding of the influence of behavior and abiotic factors on reproductive success.

Contrary to our hypothesis, breeding season weather terms (breeding season storms, temperature, and precipitation) were not strong predictors of reproductive success. This is consistent with findings by Finch et al. (2014), suggesting that carry‐over effects of temperature and precipitation in winter and spring influenced breeding phenology and brood size more than breeding season weather in three migratory passerines. However, Ockendon et al. (2013) observed that breeding season temperature and precipitation were more important predictors of breeding phenology than winter temperature and precipitation. In the case of black‐bellied plovers, breeding season weather may have limited impact on reproductive success because weather conditions in the Arctic are not always directly related to food availability (Saalfeld et al., 2019).

Consistent with our hypotheses, we found that migration duration and to a lesser extent number of stopovers were moderate predictors of reproductive success. This suggests that the effects of individual decisions pertaining to migration strategy that accumulate to determine the migration duration and number of stopovers are more influential toward reproductive success than breeding season weather conditions, at least among our tracked sample. Based on our model, a shorter migration with fewer stopovers resulted in increased probability of reproductive success. This makes sense from the perspective of efficiency; ideally, birds would minimize time and energy spent on migration, and they have evolved strategies to balance energy expenditure and acquisition (Alerstam & Lindström, 1990). Faster migration has been linked to stopover quality and subsequent reproductive success (Gómez et al., 2017), and our results are consistent with this. A longer migration with more stopovers may mean that birds are less able to accumulate the energy they need at each step of the migration (Prop et al., 2003). In addition to the balance of energy expenditure and acquisition, fewer stopovers and a shorter migration duration may indicate that fewer problems (such as storms, disturbances) were encountered during migration, or that the stopover sites chosen were of high quality (Roques et al., 2021). However, the influence of migration metrics on reproductive success can be expected to be different in some systems that are different than migratory wetland‐dependent shorebirds such as black‐bellied plovers; for example, birds with shorter migrations or migrations not constrained by wetland habitat requirements (e.g., Buchan et al., 2021).

The strong influence of weather during both migration and nonbreeding on our migration metrics suggests the presence of fitness consequences of weather within and across seasons. These consequences appear in migration decision‐making which results at least partially from weather conditions birds experienced, and propagated to explain variation in reproductive success. Migration duration results from the accumulation of decisions related to migration phenology, and phenology can often be explained by winter and spring weather conditions (Cotton, 2003; Haest et al., 2020; Van Buskirk et al., 2009), so it is not surprising that NDVI, nonbreeding temperature, nonbreeding precipitation, and migration temperature all explained substantial variation in it.

Although migration duration and the number of stopovers were not strongly correlated with one another, they were similarly influenced by nonbreeding temperature, migration temperature, and NDVI. Higher temperatures increase food availability and could allow birds to refuel more efficiently before and during migration (Evans, 1976). NDVI can influence migration phenology (Tøttrup et al., 2010), so also likely influences the decisions resulting in phenology. If we assume that NDVI is a proxy for invertebrate availability (Buchan et al., 2021; Pettorelli et al., 2005) for black‐bellied plovers, higher NDVI during migration could reduce the amount of time needed to acquire necessary energy, resulting in the reduced duration of migration that we observed. Together, these features could mean the quality of stopovers were generally higher and less stops were needed, but further research on food availability would be required to quantify habitat quality in relation to NDVI.

Additionally, as we predicted, an increase in extreme weather increased the number of stopovers, which we believe is because birds experienced increased energy requirements or less ability to move in poor weather conditions. Migration during extreme weather is physically and physiologically challenging (Gardner et al., 2017; van den Boreke & Gunkel, 2021) and therefore likely to increase the energy requirement of migration. Cumulative precipitation in the months preceding the migration period influenced the number of stopovers more than precipitation in the days leading up to stopover site use. Surface water is very dynamic and causes shorebird distribution and use to also be dynamic (Skagen & Knopf, 1994; Steen et al., 2018). Although the connection between precipitation rate and surface water is complicated, it may be that short‐term variation in precipitation causes less variation in water on the landscape than long‐term precipitation conditions do. Increased precipitation at stopovers can be expected to increase fitness; variation in shorebird abundance at stopovers have been linked to precipitation and drought (Anderson et al., 2021; Steen et al., 2018), suggesting that food abundance brought about by precipitation could also influence shorebird survival. In our study, greater precipitation resulted in fewer stopovers, so this may mean that birds stopping in areas with more moisture already in the soil do not need as many stops to obtain the necessary energy due to moist‐soil conditions leading to more productive temporary wetlands.

Broad‐scale migration metrics, especially migration duration, are the product of decisions made related to migration timing. Further studies of phenology would be beneficial to understanding the carry‐over effects of weather on productivity in migratory shorebirds. Additionally, stopover habitat quality is undoubtably important for black‐bellied plover migration, so subsequent investigations on the influence of landscape characteristics on stopover habitat selection would provide a more complete understanding of the influence of both weather and habitat quality on migration metrics.

Although there were limitations to our ability to identify fledging, our estimate of 50% of individuals reaching full‐term incubation using location data is similar to the estimate by Hussell and Page (1976) who found that 54.7% of black‐bellied plover nests reached a full‐term incubation using on‐the‐ground observations. Assuming that reproductive success rates have not changed significantly over time, this suggests that our approach worked well for identifying successful incubation. The tracking devices we used limited our inference on reproductive success to a full‐term incubation. However, it is possible using a tracking device integrating both GPS and acceleration to identify successful breeding attempts, failed breeding attempts, and breeding deferrals (Cunningham, 2019; Schreven et al., 2021). The ability to identify deferrals would allow the decision to breed, which is likely the result of physiological constraints, to be linked with conditions in previous seasons. It is also important to note that all of the tagged birds in this study were male, and there are known differences between male and female behavior in black‐bellied plovers. Males arrive earlier to breeding areas and depart later than females (Poole et al., 2020). Males are expected to be less physiologically constrained at the beginning of the breeding season because they do not lay eggs, but more physiologically constrained at the end of the breeding season because the female leaves immediately after hatching and leaves all care of the young to the male (Poole et al., 2020).

The influence of carry‐over and cross‐seasonal effects of weather and landscape conditions on fitness and phenology in birds has been well‐established in the literature (e.g., Duriez et al., 2012; Rockwell et al., 2012; Sedinger & Alisauskas, 2014; Sutton et al., 2021). However, without rich individual‐level data (e.g., GPS information collected several times per day for months), it is difficult to disentangle the mechanisms through which weather is influencing phenology or fitness. Our study revealed complex linkages among weather, migration strategy, and reproductive success in black‐bellied plovers. Yet extreme weather, droughts, and sea level rise are likely to become more problematic in future years, resulting in further loss and degradation of habitat (Piersma & Lindström, 2004). These changes are likely to constrain birds, which we anticipate will make individual decision‐making during migration more important. We encourage cross‐flyway and multispecies assessments of individual decision‐making throughout the annual cycle for a more comprehensive assessment of shorebird responses to land use and climate change. In particular, further monitoring of bird responses to winter and stopover conditions, which are particularly under‐studied, is critical for discovering drivers of shorebird migration strategies and reproductive success and optimizing conservation plans.

## AUTHOR CONTRIBUTIONS

**Sarah J. Clements:** Conceptualization (equal); data curation (equal); formal analysis (lead); funding acquisition (equal); investigation (equal); methodology (lead); project administration (equal); validation (lead); visualization (lead); writing – original draft (lead); writing – review and editing (equal). **Jason P. Loghry:** Data curation (equal); formal analysis (supporting); investigation (supporting); methodology (supporting); project administration (equal); writing – review and editing (equal). **Bart M. Ballard:** Conceptualization (equal); funding acquisition (equal); investigation (equal); project administration (equal); supervision (supporting); writing – review and editing (equal). **Mitch D. Weegman:** Conceptualization (equal); funding acquisition (equal); investigation (equal); methodology (equal); project administration (equal); supervision (lead); writing – review and editing (equal).

## FUNDING INFORMATION

Funding for this study was provided by a National Science Foundation Graduate Research Fellowship awarded to S.J.C., the Robert J. Kleberg, Jr. and Helen C. Kleberg Foundation, Upper Mississippi River/Great Lakes Joint Venture, University of Missouri, Texas A&M University – Kingsville, Audubon Society of Missouri, Environment and Climate Change Canada (awarded to R. Clark), Webster Groves Nature Study Society, The Waterbird Society, and the Animal Behavior Society.

## Supporting information


Appendix S1

## Data Availability

Bird location data used in this study will be archived using Movebank should this manuscript be accepted. All environmental data used are publicly available for download as described in the Methods section.
